# Prevalence of needle-stick and sharp object injuries and its associated factors among staff nurses in Dessie referral hospital Amhara region, Ethiopia, 2018

**DOI:** 10.1186/s13104-018-3930-4

**Published:** 2018-11-28

**Authors:** Ayele Mamo Abebe, Mesfin Wudu Kassaw, Nathan Estifanos Shewangashaw

**Affiliations:** 1 Department of Nursing, Debre Birhan Health Sciences College, P.O. Box 37, Amhara, Debre Birhan, Ethiopia; 2 Department of Nursing, College of Health Sciences, Woldia University, P.O. Box 400, Amhara, Weldiya, Ethiopia; 3 Public Health Department, Woldia University, P.O. Box 400, Amhara, Weldiya, Ethiopia

**Keywords:** Needle stick and sharp object injury, Nurses, Dessie referral hospital

## Abstract

**Objective:**

The aim of this study was to assess the prevalence of needle-stick and sharp object injuries among staff nurses in Dessie referral hospital, Amhara region, Ethiopia, 2018.

**Results:**

Among the 151 study participants, 98 (65%) respondents were males. Seventy-five (48.1%) participants had 4–10 years of experience. The overall prevalence of needle stick and sharp object injury among staff nurses in Dessie referral hospital was 43%. In this study, nurses who worked in the emergency department were 11× more likely to experience needle stick and sharp object injury compared with nurses who worked in outpatient department P = 0.004 [AOR = 11.511 95% CI 2.134, 62.09)]. Participants who were worked in adult health department were 10× more likely experience needle stick and sharp object injury when compared with participants who were worked in outpatient department P = 0.006 [AOR = 9.742 95% CI 1.904, 49.859)]. The major implication of these study findings on the health system is the importance of given emphasis for nurses in relation with needle stick and sharp injury.

**Electronic supplementary material:**

The online version of this article (10.1186/s13104-018-3930-4) contains supplementary material, which is available to authorized users.

## Introduction

A needle stick injury is a penetration of skin by a needle point, but probably also by other piercing instruments [1]. These problems are a common event in the healthcare environment. These injuries also commonly occur during drawing blood, medication administering, needle recapping and during surgery [2].

In a report by Center for Disease Control and Prevention there is an estimated 600,000 to one million needle stick injuries occurring each year in the world, and about half of which went unreported [3]. Findings of researches on needle stick injury showed that it ranged from 21 to 95% [4–6]. Needle stick injury has accounted for 86% of all occupationally related infection transmissions [7].

Globally, two million health care workers suffer from accidental needle stick injury each year [8]. In UK, a study showed that 37% needle stick injuries reported at some stage during their career [9]. In Nepal a survey reported that needle stick injury among health care workers was 70.3% [10].

In developing countries, where the prevalence of HIV infection is the highest in the world, the number of needle stick injuries is also the highest. Additionally, African health care workers suffer an average of two to four needle stick injuries per year and physicians are much less likely to report a needle stick injury than other healthcare professionals [11]. Study revealed that, transitional and developing countries where unnecessary injection is common, the average number of health care injection per person is averagely estimated to be 3.7/year. Generally in developing countries exposure and health impact of NSI are rarely monitored and poorly managed [12].

In Turkey study, 44.3% of the nurse experience a sharp or needle sticks injury during their professional life. These injuries happened persistently when the nurses were removing a needle from rubber or other tough material; recapping a used needle and take apart a device or equipment [13].

In other studies, two-third healthcare workers in hospitals were injured by sharp or needle sticks [14, 15]. Furthermore, studies report from Addis Ababa, Arba Minch, Bahir Dar and Awi Zone indicated occupational exposure to needle stick and sharp injuries among HCWs were 66.6%, 42.1%, 18.7% and 31.0%, respectively [16–19].

In a national survey of Ethiopia, 38% of health facilities were removed the sharps objects and needles in an open ground [20]. Similarly in other study, 35% of the health facilities were disposed the dirty syringes and needles in a way that exposed the health workers and the community for injury [21].

For many years, Ethiopia government gave health education on standard precautions and many preventions methods to avoid sharp and needle sticks injuries but, the problem is not decreased.

Therefore, the study aimed to assess the prevalence and associated factors to needle stick and sharp injury among nurses at Dessie referral hospital, Amhara, Ethiopia.

## Main text

### Methods

#### The study area and period

This study was conducted in Dessie referral hospital, Amhara region; Northeast Ethiopia from February 1–15, 2018. Dessie is the capital city of south Wollo zone and is located in the North eastern part of Ethiopia, at approximately 401 km from the capital city, Addis Ababa. There were 256 nurses who are working in Dessie referral hospital. The source population and study population was all staff nurses in Dessie referral hospital, Amhara region, Ethiopia.

#### Study design

The study design was an institutional based cross sectional study.

#### Sample size

To determine the sample size for the study, the following assumptions were considered:

- P = 31% [16]
- 5% margin of error (d = 0.05)
- 10% for non-response rate.

The sample size was calculated by using the formula:

$${\text{n}}_{\text{i}} = \frac{{{\text{z}}^{2} {\text{p}}(1 - {\text{p}})}}{{{\text{d}}^{2} }}$$

where n = sample size

$$\begin{aligned} {\text{n}}_{\text{i}} = & (1.96) \times (1.96) \times 0.31(0.69)/(0.05)(0.05)\, \text{for population} \\ & = 328 \,({\text{I}}\;{\text{use}}\;{\text{this}}\;{\text{sample}}\;{\text{size}}\;{\text{as}}\;{\text{it}}\;{\text{is}}\;{\text{when}}\;{\text{the}}\;{\text{study}}\;{\text{population}}\;{\text{was}}\;{\text{greater}}\;{\text{than}}\; 10, 000)\\ \end{aligned}$$

But our study population 256 is less than 10,000 the calculation. So, we were used the correction formula to get final sample size, i.e. $nf = {\text{n}}_{\text{i}} /\left( {1 + \frac{{{\text{n}}_{\text{i}} }}{N}} \right) = 328/\left( {1 + \frac{328}{256}} \right)$ = 144, and we added non-respondent rate of 10%.

Therefore final sample size was 158.

#### Sampling procedure

**Fig. 1:**
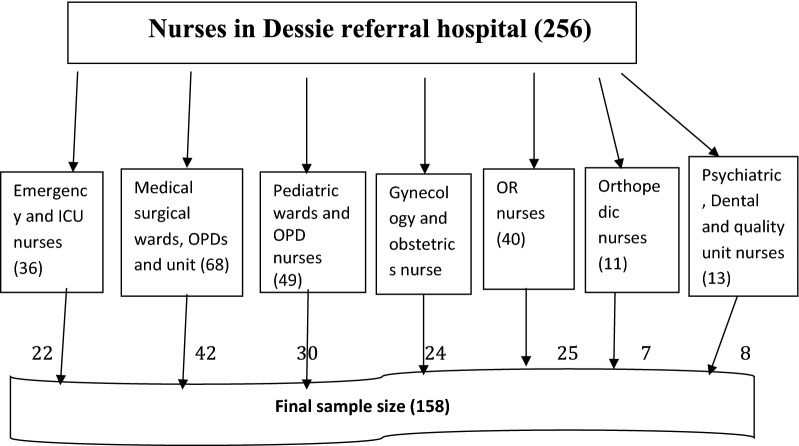
Sampling procedure of participants in Dessie referral hospital, 2018

All 256 nurses working in Dessie referral hospital were considered for the study. Participants were selected by using simple random sampling technique from each department based on proportion until the required sample size obtained (Fig. 1).

#### Data collection procedure

Self-administered structured questionnaires were used to collect the data. The questionnaires were taken from different related studies [16, 18, 19] and necessary modifications were done. A translated Amharic version questionnaire was used to collect data. Four health extension workers for data collectors and two B.Sc. nurses for supervisor were employed.

#### Data quality control

Training was given for data collectors and supervisors for 1 day and the questionnaire pretested on 5% sample size at Boru Meda hospital. The questionnaires was checked by the principal investigators on daily basis for completeness.

#### Data processing and analysis

Data were checked for completeness and consistency by principal investigators before data entry. Completed data were entered and coded by number into a commuter software Epi data statistical package and exported to SPSS version 20. Bivariate analysis with a P-value < 0.05 was employed to select candidate variables for the multivariable logistic regression. All statistical tests were considered significant if P-value < 0.05. Finding was presented by using frequency, tables and graphs.

### Results

#### Socio demographic characteristics of study participants

In this study, 151 participants were participated in the study with the response rate of 95.6%. Among the 151 study participants, 98 (65%) participants were males. Majority of the respondents (104 (69.0%)) were BSc holders. Seventy-five (48.1%) participants had 4–10 years of experience (Table 1).

**Table 1:** The socio-demographic characteristics of participants, Dessie referral hospital in 2018 (n = 151)

Variables	Frequency (n = 151)	Percent (%)
Gender		
Male	98	65.0
Female	53	35.0
Age		
18–24	40	25
25–30	51	36
31–35	36	23.1
36–40	14	9
> 41	10	6.5
Marital status		
Single	72	46.7
Married	79	52.3
Educational status		
Diploma	46	30.4
B.sc.	104	69.0
M.sc.	1	0.6
Work experience (years)		
<3	60	39.0
4–10	75	48.1
11–15	16	9.0
> 15	7	3.9

B.sc., Bachelor of Science; M.sc., Master of Science

#### Work and behavioral factors related characteristics of participants

Out of the total participants, 49 (32%) participants were from emergency department. Majority of the study participants (104 (69.0%)) had dissatisfied with their job (Table 2).

**Table 2:** Work related and behavioral characteristics of participants

Variables	Frequency (n = 151)	Percent (%)
Working department		
Emergency	49	32
Pediatric word	10	6.5
Adult ward	28	18.5
OPD, ICU, orthopedic and others	65	43
Job satisfaction		
No	47	31
Yes	104	69
Number of injection per day		
5–10	7	4.5
10–15	12	8
15–20	37	24.5
> 20	95	63
Presence of Infection prevention committee		
No	32	21.0
Yes	119	79.0
Vaccination of HBV		
No	70	46.3
Yes	81	53.7

*OPD* outpatient department; *ICU* intensive care unit

#### Information related to needle stick and sharp object injury

Among the total participants, 89 (59%) of them reported that there was no post exposure management facility and 72 (42.6%) respondents didn’t know which unit they should report when they face needle stick and sharp object injury (Additional file 1: Table S1).

#### Frequency distribution of type of injection use, type of injury and occasion of injury occurrences

In relation with the type of needle stick and sharp object injury, 15% of respondents were reported they faced deep needle stick and sharp object injury while 56% of respondents faced slight skin penetration (Additional file 2: Figure S1).

#### Frequency distribution medical devices during needle stick injury

In this study, syringe with needle (e.g. medication needles) was the device that caused more injury (31%), followed by suturing needle (30.2%) (Additional file 3: Figure S2).

#### Frequency distribution of needle, syringes and sharps disposal methods

Based on this study, protected incineration (76%) was the commonest disposable method with followed by open dumping (12%) and open incineration (8%) (Additional file 4: Figure S3).

#### The prevalence needle stick and sharp injury

The overall prevalence of needle stick and sharp object injury among staff nurses in Dessie referral hospital was 43% (Additional file 5: Figure S4).

#### Factors associated with needle stick and sharp object injury

In this study, nurses who worked in emergency department were 11× more likely to experience needle stick and sharp object injury compared with nurses who worked in outpatient department P = 0.004 [AOR = 11.511 95% CI 2.134, 62.09)]. Participants who were worked inpatient department were 10× more likely experience needle stick and sharp object injury when compared with participants who were worked in outpatient department P = 0.006 [AOR = 9.742 95% CI 1.904, 49.859)]. Participants who had training on needle stick and sharp object injury were 4× less likely to experienced needle stick and sharp object injury as compared with those who had not training P = 0.021 [AOR = 3.818, 95% CI 1.221–11.935)]. In this study, nurses who practice recap of needle were 4× more likely to experience needle stick and sharp object injury when compared with nurses who didn’t practice needle recap [AOR = 4.344, 95% CI (1.186, 15.906)]. Participants who were not apply universal precaution were 6× more likely to experienced needle stick and sharp object injury as compared with those who were apply universal precaution P = 0.001 [AOR = 6.413, 95% CI 2.072–19.850)]. (Additional file 6: Table S2).

### Discussion

According to this study, the prevalence of needle stick and sharp object injury in Dessie referral hospital was 43%. This finding was align with many international studies [6, 10, 13]. On the other hand, the current study finding was higher than the finding of studies done in Malawi (30.3%), Nigeria (23.1%), and India (30.1%) [12, 19, 21] and lower than the studies done in Egypt (62.3%), Nigeria (68.3%), Sarajevo, Bosnia and Herzegovina (61.1%), and Saudi Arabia (50.9%), [4, 5, 7, 11]. The prevalence of needle stick and sharp object injury reported from the studies conducted in Addis Ababa, Arba Minch, Bahir Dar, Awi were 66%, 42%, 33% and 18.7%, respectively [14–17]. The variation of these findings may be due to the difference of study design used, the socio demographic, and cultural characteristics of study participants. Also it could be due to the difference in the study health facility setups; even the year of the study.

Compared to other groups of health workers, it can be assumed that nurses are more likely to experience needle stick and sharp object injury, because of their working condition. Comparing this study results with a facility based cross sectional study conducted in Awi zone, we found our rates to be higher across the board; nurses vs physician (43% vs 16%), nurses vs health officer (43% vs 16.7%), nurses vs laboratory (43% vs 11.1%,), and nurses vs midwives (43% vs 25%) [17]. The higher magnitude in among nurses might reflect the particular occupational health hazard experienced by this group of health workers.

In this study, nurses who worked in emergency department were 11× more likely to experience needle stick and sharp object injury compared with nurses who worked in Outpatient department P = 0.004 [AOR = 11.511 95% CI 2.134, 62.09)]. The finding was consistent with studies done in Ethiopia, and Nigeria [12, 14].

According to this study, nurses who were worked inpatient department were 10× more likely experience needle stick and sharp object injury when compared with participants who were worked in outpatient department P = 0.006 [AOR = 9.742 95% CI 1.904, 49.859)]. The finding was in line with studies done in Nigeria and in Awi zone in Ethiopia, [14, 17].

Unlike the study conducted in Arba Minch general hospital, Ethiopia [15], study subjects who had training on needle stick and sharp object injury were 4× less likely to experienced needle stick and sharp object injury as compared with those who had not training in this study. The difference may be more education and training might be given about needle stick injury and infection prevention in this study. Education and training is the right tools to bring behavioral change about infection prevention and safety measures.

In this study, nurses who practice recap of needle were 4× more likely to experience needle stick and sharp object injury when compared with nurses who didn’t practice needle recap [AOR = 4.344, 95% CI (1.186, 15.906)]. It was in line with the studies conducted in Ethiopia, America and Philippines which showed that recap of needle is a risk factor for needle stick and sharp injury [2, 3, 10, 20].

Based on this study, nurses who were not apply universal precaution were 6× more likely to experienced needle stick and sharp object injury as compared with those who were apply universal precaution P = 0.001 [AOR = 6.413, 95% CI 2.072–19.850)]. This study is in line with the study done in Awi Zone [17].

### Conclusions

In this study, prevalence of needle stick and sharp object injury among staff nurses in Dessie referral hospital was 43%. Apply universal precaution, working department, training on needle stick and sharp object injury and recap of needle had shown a significant association with needle stick and sharp object injury. Education and training should be given for nurses on this issue to reduce this problem.

## Limitations

Cross sectional study design had no shown cause-effect relationship (Chicken-eggs dilemma). It would be better if qualitative approach study was triangulated to investigate further factors on needle stick and sharp object injury.

## Additional files

**Additional file 1: Table S1.** Information participants to needle stick and sharp object injury, 2018.

**Additional file 2: Figure S1.** Frequency distribution of type of injection use, type of injury and occasion of injury occurrences, Dessie referral hospital, Amhara region, 2018.

**Additional file 3: Figure S2.** Frequency distribution medical devices during needle stick injury among nurses in Dessie referral hospital, Amhara region, 2018.

**Additional file 4: Figure S3.** Frequency distribution of needle syringes and sharps disposal in Dessie referral hospital, 2018.

**Additional file 5: Figure S4.** Prevalence of needle stick and sharp injury among nurses in Dessie referral hospital, 2018.

**Additional file 6: Table S2.** Bivariate and multivariate logistic regression analysis of factors associated with needle stick and sharp object injury in Dessie referral hospital, Ethiopia 2018.

## Abbreviations

HBV: hepatitis B virus
HCV: hepatitis C virus
HCW: health care workers
HIV: human immunodeficiency virus
NSI: needle stick injury
PEP: post exposure prophylaxis
ICU: intensive care unit
