# Supplementary material for: Prevalence of needle-stick and sharp object injuries and its associated factors among staff nurses in Dessie referral hospital Amhara region, Ethiopia, 2018
Source: BMC Res Notes. 2018 Nov 28;11:840. doi: 10.1186/s13104-018-3930-4 (PMC6263535; doi:10.1186/s13104-018-3930-4)
Supplement: Supplementary file 1 — Additional file 1: Table S1. Information participants to needle stick and sharp object injury, 2018. [file 13104_2018_3930_MOESM1_ESM.docx]

Table S1: - Information related on prevention of needle stick and sharp object injury, 2018.

| **Variables** | **Frequency** | **Percent (%)** |
| --- | --- | --- |
| **Presence of post exposure management facility** |  |  |
| Yes | 62 | 41.06% |
| No | 89 | 59.9% |
| **Do you know which unit you may report needle stick injury** |  |  |
| Yes | 79 | 52.4% |
| No | 72 | 47.6% |
| **Is there enough privacy during counseling and diagnosis staff face NSI** |  |  |
| Yes | 19 | 12.5% |
| No | 132 | 87.5% |
| **Had training on IP** |  |  |
| Yes | 43 | 28.4% |
| No | 98 | 71.6% |
| **Recapped of needle** |  |  |
| Yes | 36 | 23.8% |
| No | 125 | 76.2% |
| **How the condition recap** |  |  |
| One hand | 27 | 75% |
| Two hand | 9 | 35% |
| **Observe reuse of needle** |  |  |
| Yes | 7 | 4.6% |
| No | 144 | 95.4% |
| **How is injection Environment** |  |  |
| Clean and no potential contamination | 42 | 27.8% |
| Dirty and presence of potential communication | 109 | 72.2% |
| **Presence of sharp collection box injection room** |  |  |
| Yes | 133 | 88% |
| No | 18 | 12% |
| **Presence of sharps in place where expose health workers to NSI** |  |  |
| Yes | 65 | 43% |
| No | 86 | 57% |
| **Have you ever seen sharp container which is over filled or torn** |  |  |
| Yes | 79 | 52.3% |
| No | 72 | 47.7% |
| **Follow universal precaution** |  |  |
| Yes | 60 | 39.7% |
| No | 91 | 60.3% |
| **Availability of safety box** |  |  |
| Yes | 126 | 83.4% |
| No | 25 | 16.6% |
