# Supplementary material for: Prevalence of needle-stick and sharp object injuries and its associated factors among staff nurses in Dessie referral hospital Amhara region, Ethiopia, 2018
Source: BMC Res Notes. 2018 Nov 28;11:840. doi: 10.1186/s13104-018-3930-4 (PMC6263535; doi:10.1186/s13104-018-3930-4)
Supplement: Supplementary file 6 — Additional file 6: Table S2. Bivariate and multivariate logistic regression analysis of factors associated with needle stick and sharp object injury in Dessie referral hospital, Ethiopia 2018. [file 13104_2018_3930_MOESM6_ESM.docx]

Table S2:-Bivariate and multivariate logistic regression analysis of factors associated with needle stick and sharp object injury in Dessie referral hospital, Ethiopia 2018.

| **Variables** | **NSI** | | **OR (95% CI)** | |  |
| --- | --- | --- | --- | --- | --- |
|  | **Yes** | **No** | **COR** | **AOR** | **P-Value** |
| **Gender** |  |  |  |  |  |
| Female | 33 | 20 | 3.403(1.107-8.637) | 4.819(.290-80.082) | .273 |
| Male | 32 | 66 | 1 | 1 |  |
| **Department** |  |  |  |  |  |
| Adult in patient care | 15 | 13 | 9.565(1.096-15.910) | **9.742(1.904-49.859) *** | **.006** |
| Pediatric | 6 | 4 | 12.24(1.896-16.675) | 5.710(.824-39.559) | .078 |
| Emergency | 37 | 11 | 27.87(3.867-29.674) | **11.511(2.134-62.09) *** | **.004** |
| OPD | 7 | 58 | 1 | 1 |  |
| **Satisfaction with Job** |  |  |  |  |  |
| Yes | 28 | 19 | 2.669(1.468-5.963) | .221(.012-4.031) | .309 |
| No | 37 | 67 | 1 | 1 |  |
| **Injection Environment** |  |  |  |  |  |
| Clean and no contamination | 32 | 10 | 7.370 (1.019-8.345) | .782(.188-3.243) | .734 |
| Contaminated | 33 | 76 | 1 | 1 |  |
| **Training on needle stick injury** |  |  |  |  |  |
| Yes | 38 | 15 | 6.662 (1.094-7.848) | **3.818(1.221-11.935) *** | **.021** |
| No | 27 | 71 | 1 | 1 |  |
| **Availability of safety box** |  |  |  |  |  |
| No | 18 | 7 | 4.322 (1.152-5.767) | 2.608(.681-9.995) | .162 |
| Yes | 47 | 79 | 1 | 1 |  |
| **Number of injection per day** |  |  |  |  |  |
| >20 | 69 | 26 | 6.635(1.865-7.98) | .207(.020-2.186) | .190 |
| 15-20 | 9 | 28 | .804(.235-.904) | .092(.009-1.020) | .052 |
| 10-15 | 6 | 6 | 2.500(1.066-5.588) | .841(.069-10.282) | .892 |
| 5-10 | 2 | 5 | 1 | 1 |  |
| **Recap needle** |  |  |  |  |  |
| Yes | 29 | 7 | 9.091 (1.253-10.643) | **4.344(1.186-15.906) *** | **.027** |
| No | 36 | 79 | 1 | 1 |  |
| **Apply universal precaution** |  |  |  |  |  |
| No | 42 | 18 | 6.899(1.035-8.116) | **6.413(2.072-19.850) *** | **.001** |
| Yes | 23 | 68 | 1 | 1 |  |

**Abbreviation: ETB:** Ethiopian Birr**, *statistically significant at P< 0.05, OPD:** Out Patient Department**, ICU:** Intensive Care Unit
