# Supplementary material for: Prevalence of needle-stick and sharp object injuries and its associated factors among staff nurses in Dessie referral hospital Amhara region, Ethiopia, 2018
Source: BMC Res Notes. 2018 Nov 28;11:840. doi: 10.1186/s13104-018-3930-4 (PMC6263535; doi:10.1186/s13104-018-3930-4)
Supplement: Supplementary file 3 [file 13104_2018_3930_MOESM2_ESM.docx]

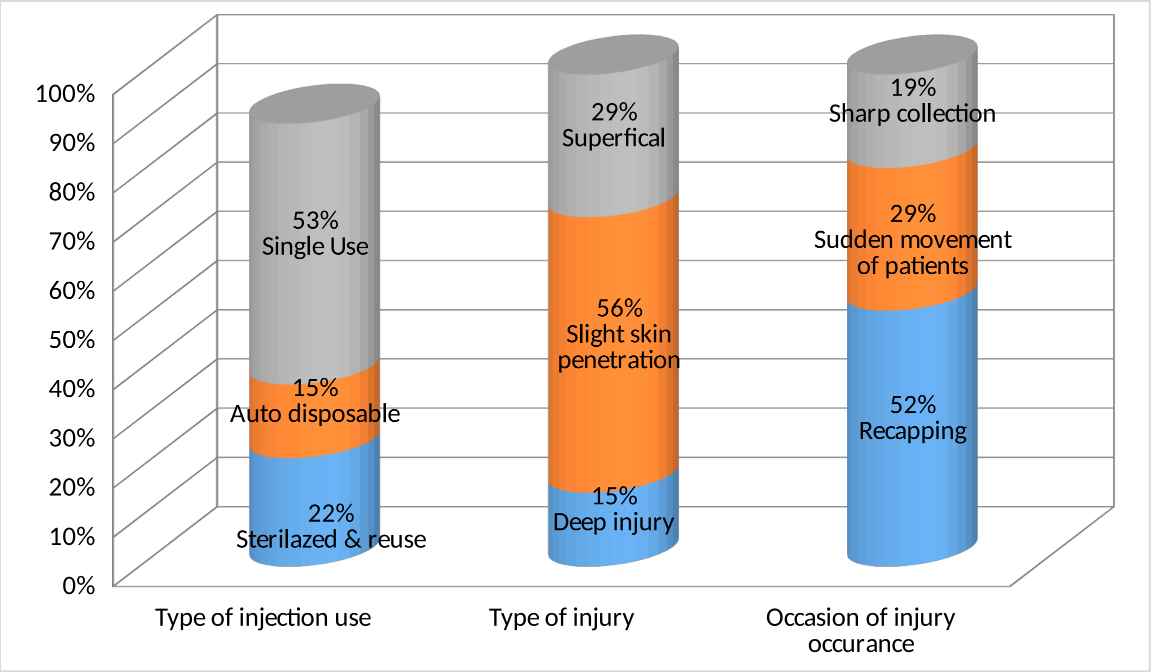
100%
90%
80%
70%
60%
50%
40%
30%
20%
10%
0%
53%
Single Use
15%
Auto disposable
22%
Sterilazed & reuse
29%
Superfical
56%
Slight skin penetration
15%
Deep injury
19%
Sharp collection
29%
Sudden movement of patients
52%
Recapping
Type of injection use
Type of injury
Occasion of injury occurance
