# Supplementary material for: Prevalence of needle-stick and sharp object injuries and its associated factors among staff nurses in Dessie referral hospital Amhara region, Ethiopia, 2018
Source: BMC Res Notes. 2018 Nov 28;11:840. doi: 10.1186/s13104-018-3930-4 (PMC6263535; doi:10.1186/s13104-018-3930-4)
Supplement: Supplementary file 4 [file 13104_2018_3930_MOESM3_ESM.docx]

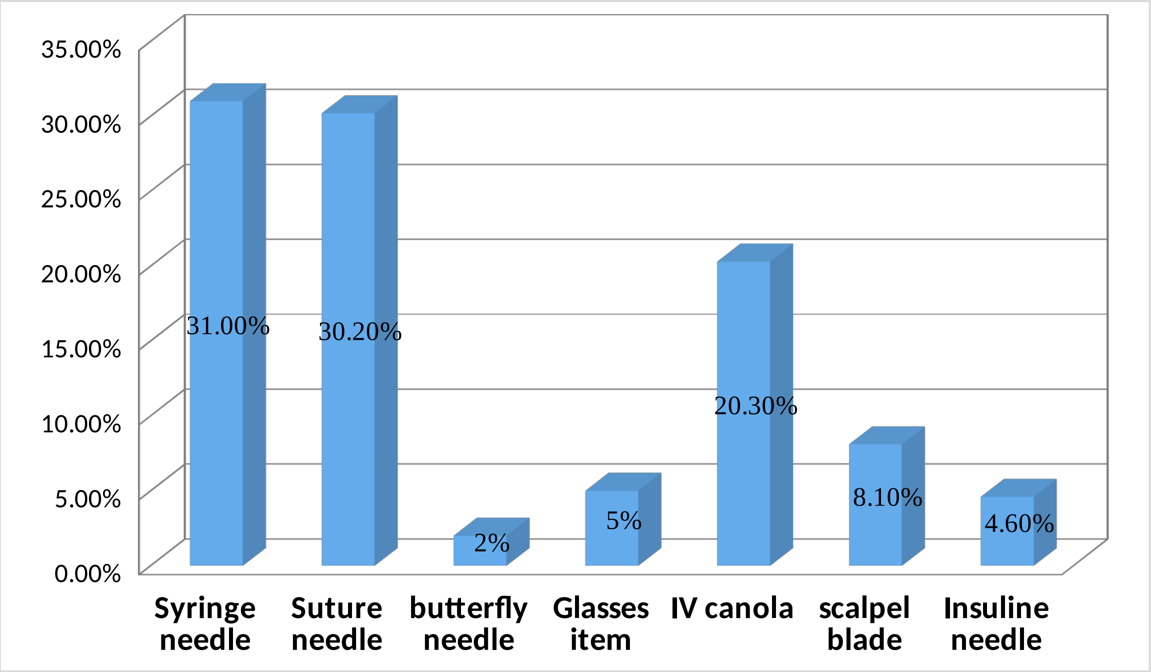
35.00%
30.00%
25.00%
20.00%
15.00%
10.00%
5.00%
0.00%
31.00%
30.20%
2%
5%
20.30%
8.10%
4.60%
Syringe needle
Suture needle
butterfly needle
Glasses item
IV canola
scalpel blade
Insuline needle
