# Supplementary material for: Prevalence of needle-stick and sharp object injuries and its associated factors among staff nurses in Dessie referral hospital Amhara region, Ethiopia, 2018
Source: BMC Res Notes. 2018 Nov 28;11:840. doi: 10.1186/s13104-018-3930-4 (PMC6263535; doi:10.1186/s13104-018-3930-4)
Supplement: Supplementary file 6 [file 13104_2018_3930_MOESM5_ESM.docx]

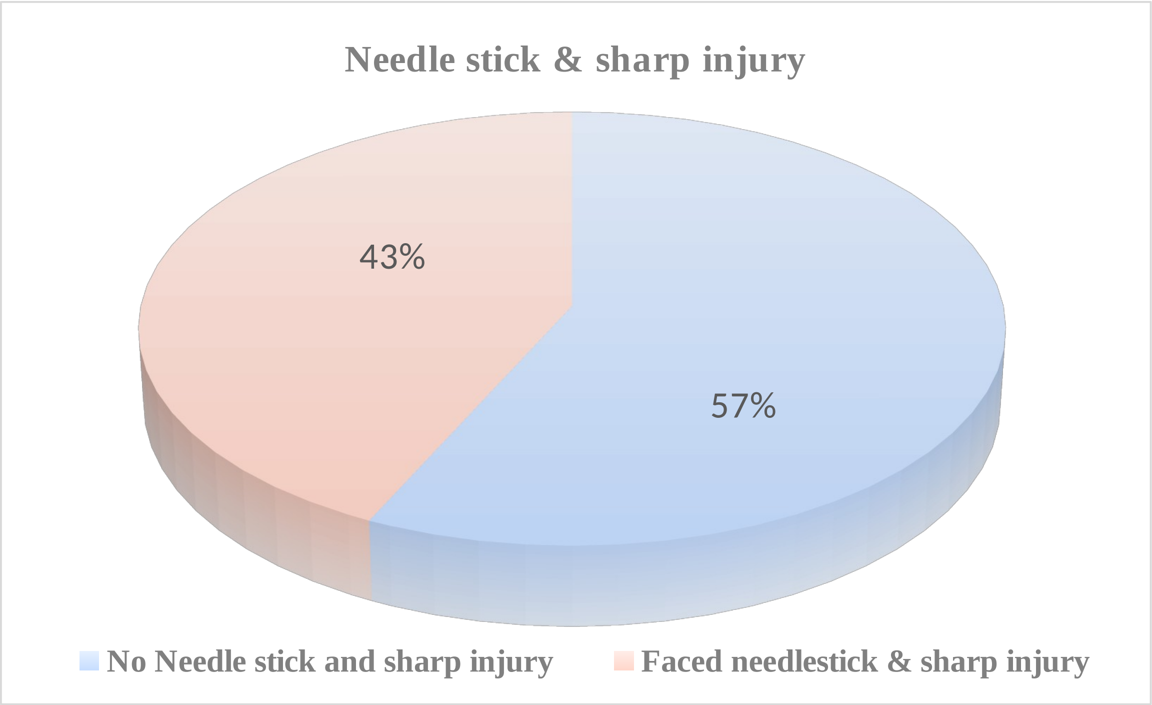
Needle stick & sharp injury
43%
57%
No Needle stick and sharp injury
Faced needlestick & sharp injury
